# Medium- to long-term outcomes of vaginally assisted laparoscopic sacrocolpopexy in the treatment of stage III–IV pelvic organ prolapse

**DOI:** 10.1186/s12905-022-02105-1

**Published:** 2022-12-07

**Authors:** Tuo Deng, Su Wang, Xuezao Liang, Liquan Chen, Yanli Wen, Xiaowei Zhang, Lizhen Xu

**Affiliations:** 1 Female Pelvic Floor Unit, Department of Urology, The First Affiliated Hospital of Guangzhou Medical University, Guangzhou Medical University, Kangda Road 1#, Haizhu District, Guangzhou, 510230 Guangdong China; 2 Guangdong Key Laboratory of Urology, The First Affiliated Hospital of Guangzhou Medical University, Guangzhou Medical University, Guangzhou, China; 3 Department of Obstetrics and Gynecology, The First Affiliated Hospital of Guangzhou Medical University, Guangzhou Medical University, Guangzhou, China; 4 Department of Obstetrics and Gynecology, The First People’s Hospital of Foshan, Foshan, China; 5 Department of Obstetrics and Gynecology, Guangzhou First People’s Hospital, Guangzhou, China

**Keywords:** Efficacy, Medium- to long-term outcomes, Pelvic organ prolapse, Safety, Vaginally assisted laparoscopic sacrocolpopexy

## Abstract

**Background:**

Vaginally assisted laparoscopic sacrocolpopexy (VALS) refers to the placement of synthetic meshes through the vagina in addition to traditional laparoscopic sacrocolpopexy. In this study, we aimed to investigate the medium- to long-term efficacy and safety of VALS for treating stage III–IV pelvic organ prolapse (POP).

**Methods:**

The study was designed as a case series at a single center. Patients with stage III–IV POP in our hospital from January 2010 to December 2018 were included. Perioperative parameters, objective and subjective outcomes, and complications were assessed.

**Results:**

A total of 106 patients completed the follow-up and were included in our study. Within a median follow-up duration of 35.4 months, the objective cure ratio of VALS reached 92.45% (98/106), and the subjective success rate was 99.06% (105/106). Patients reported significant improvements in subjective symptoms. In eight patients suffering anatomic prolapse recurrence, two posterior POP cases were treated by posterior pelvic reconstruction surgery, while six anterior POP cases did not need surgical therapies. The reoperation rate was 1.89% (2/106). No intraoperative complications occurred. Three patients (2.83%) had postoperative fever, and one (0.94%) had wound infection during hospitalization. Six patients (5.66%) had mesh exposure on the vaginal wall, and de novo urinary incontinence occurred in two patients (1.89%) during the follow-up period.

**Conclusion:**

VALS is an effective and safe surgical method for treating severe POP. Therefore, VALS should be considered in the treatment of severe POP due to its favorable subjective and objective outcomes, relatively low rate of infection and acceptable rate of mesh exposure.

## Background

Pelvic organ prolapse (POP) is defined as the downward descent of the female pelvic organs (vagina, uterus, bladder, and/or rectum) into or through the vagina [1]. Although it is not a life-threatening disease, POP seriously reduces patients’ quality of life. Surgical treatment remains an important therapy for severe POP. The lifetime surgery risk of females with POP is close to 20% [2, 3]. Various surgical methods, such as native-tissue repair, sacrocolpopexy (SC) and transvaginal mesh (TVM), have been described in the management of POP. Among them, SC is increasingly considered a preferred surgery for uterovaginal prolapse, especially after vaginal synthetic mesh warnings were raised by the Food and Drug Administration (FDA) [4].

Several modifications have been applied to SC to increase the success rate and feasibility of surgery [5]. Since laparoscopic sacrocolpopexy (LSC) was first reported [6], laparoscopy has gradually become a dominant surgical procedure. However, LSC has difficulties in manipulations, and it requires high levels of experience and skill. In recent years, modified SC procedures, such as single-port laparoscopy, transvaginal natural orifice transluminal endoscopic surgery and robot-assisted LSC, have also been reported. Vaginally assisted laparoscopic sacrocolpopexy (VALS) refers to the placement of synthetic meshes through the vagina in addition to traditional LSC. von Pechmann et al. [7] reported the safety and short-term anatomic outcomes of VALS in 2011, and Aydin et al. found similar results recently [8]. A study by Nosti et al. also indicated that transvaginal placement of mesh did not increase the risk of mesh-related complications and decreased the operative time compared to transabdominal placement of mesh [9]. Nevertheless, research data on the success rate and complications of VALS in medium- to long-term follow-up periods are still lacking.

In our study, we aimed to explore the medium- to long-term efficacy and safety of VALS for treating stage III–IV POP patients through a case series at a single center. We hope our results may be helpful to provide evidence for clinical practice to some extent.

## Materials and methods

### Study design

The study was designed as a case series at a single center and followed the STROBE guidelines [10]. We included females with stage III–IV (according to the Pelvic Organ Prolapse Quantification [POP-Q] system) POP who received VALS at the First Affiliated Hospital of Guangzhou Medical University from January 2010 to December 2018. Written informed consent was obtained from participants on admission routinely. This study was approved by the Ethics Committee of the First Affiliated Hospital of Guangzhou Medical University, China. Given the design of the study, the Institutional Review Board ruled that approval was not needed.

### Demographic data collection

Baseline demographic data of eligible patients were obtained from medical records, including age at surgery, body mass index (BMI), gravidity, parity, menopausal status, comorbidity, previous surgery history, values of each POP-Q point, and POP-Q stage.

### Assessment of prolapse

The POP-Q system, including Aa, Ba, C, TVL, Ap, Bp, gh, Pb and D points, was used to assess the objective (also named anatomic) severity of prolapse. Pelvic Floor Distress Inventory-Short Form 20 (PFDI-20) [11], Pelvic Floor Impact Questionnaire Short Form 7 (PFIQ-7) [11] and Pelvic Organ Prolapse/Urinary Incontinence Sexual Questionnaire-12 (PISQ-12) [12] were administered to evaluate the subjective influence of prolapse on patients.

### Surgical indications

Surgical options for treating severe POP in our hospital included TVM, laparoscopic high uterosacral ligament suspension, laparoscopic lateral abdominal wall suspension, LSC and VALS. Surgical selection was made regarding the patient’s and surgeon’s choice. In general, patients with multicompartmental POP or requiring hysterectomy were recommended to receive VALS. The exclusion criteria were as follows: (1) posthysterectomy patients; (2) patients who did not undergo VALS at our center; and (3) patients who refused to be followed up.

### Preoperative preparations

All included patients were asked to wear the pessary, which helped avoid ulcers or bleeding, after they were admitted to the hospital until the operation started [13]. Estrogen ointment was topically applied for one to two weeks to improve the vaginal mucosa in case it was too thin for the operation according to our experiences. Patients were educated to keep the vulva and vagina clean through a potassium permanganate sitz bath every night after they were in the hospital before surgery.

### Surgical procedure

VALS was performed by experienced surgeons (X. Z. and L. X.). Surgical procedures are listed below. At the beginning, vaginal or laparoscopic hysterectomy is performed. Then, sacrocolpopexy is conducted using Gynemesh (Gynecare, Ethicon, Somerville, NJ, USA) or “Y” type mesh (ARTISYN; Johnson & Johnson international, c/o European Logistics Centre, Diegem, Belgium). The anterior gynemesh is cut into a 3.5 cm * 12.0 cm boot-shaped mesh with a 3 cm * 5 cm boot bottom, and the posterior gynemesh is cut into a 3.5 cm * 12.0 cm oblong-shaped mesh. Both Gynemesh and “Y” type meshes are modified according to patients’ anatomic features.

**Fig. 1:**
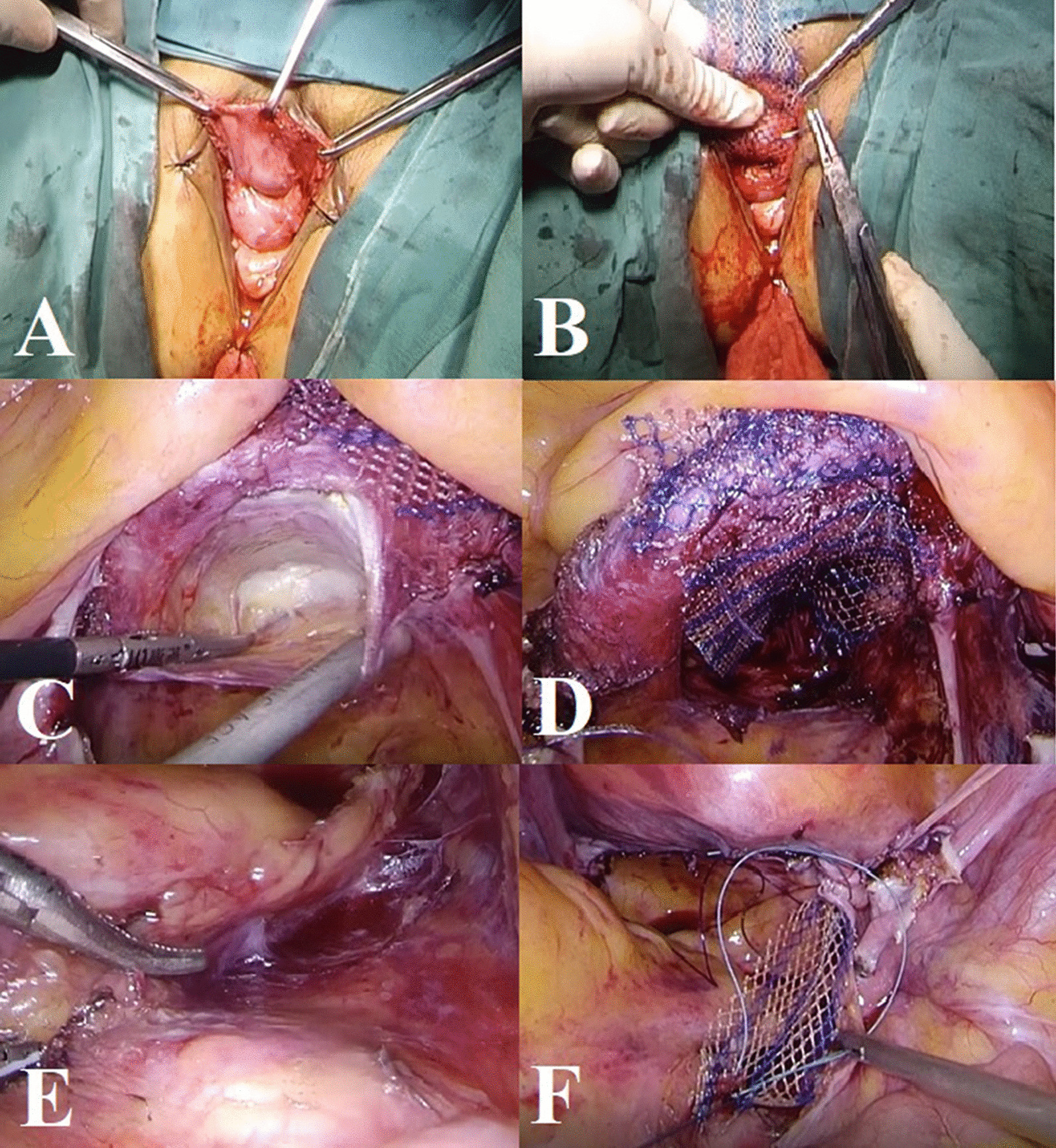
Surgical procedures of VALS: **A** dissection of anterior vaginal wall, **B** placement of anterior mesh, **C** dissection of presacral space, **D** placement of posterior mesh, **E** location of the anterior longitudinal ligament of the sacrum, **F** transfixion of mesh strips

In the sacrocolpopexy step, the vesicovaginal space is first accurately dissected from the vaginal incision left by hysterectomy to the level of the bladder neck (Fig. 1A). Second, the anterior mesh is positioned and sutured under the full thickness of the anterior vaginal wall with the distal end of the mesh reaching the level of the bladder neck using the 2-0 absorbable polyglactin 910 suture (Fig. 1B). In the following procedure, free arms of fixed meshes are placed into the abdomen, and the vaginal vault is closed with the 2-0 absorbable polyglactin 910 suture. After surgeons change gloves and set the endoscopy unit, laparoscopy will be performed, as described below. The presacral peritoneum is opened at the level of the sacrum promontory, and the presacral space is dissected up to the vaginal cuff, medially to the right uterosacral ligament and laterally to the rectum (Fig. 1C). Subsequently, the pelvic peritoneum is opened along the medial side of the right uterosacral ligament, and the upper 2/3 of the rectovaginal space is also dissected. Then, the posterior mesh is sutured to the posterior vaginal wall using 2-0 absorbable V-Loc™ (Fig. 1D). Finally, mesh strips are transfixed to the anterior longitudinal ligament of the sacrum with two separate 2-0 polypropylene sutures without tension (Fig. 1E and F). In brief, our surgical procedure is similar to that described in Athanasiou’s study [14]. The detailed procedures in our study may be different, but suturing and fixing the anterior or posterior mesh through the vagina remains the key step in VALS.

For perioperative information, we recorded concomitant surgeries, estimated blood loss, operative time of hysterectomy plus sacrocolpopexy, intra- and postoperative complications, catheterization time and hospitalization time after surgery. Complications included postoperative fever, wound infection, mesh exposure, de novo urinary incontinence, and pain during intercourse. Early complications were defined to occur during the surgery or within 4 weeks postoperatively, while late complications were defined as any complications after 4 weeks during the entire follow-up period.

### Postoperative follow-up

Patients were telephoned to return to for outpatient follow-up from November 2016 to May 2019. Patients were routinely followed up 1 month and 3 months postoperatively, and then they were asked to return every 6 months. Objective success was defined as the lowest point of prolapse never reaching the level of the hymen (point 0) [15, 16]. Subjective success was regarded as “very much improved” or “much improved” in patients’ responses by the Patient Global Impression of Improvement (PGI-I) [17]. The PFDI-20, PFIQ-7 and PISQ-12 were also used to quantify subjective outcomes. Recurrence and complications were checked in the follow-up duration. According to objective success, recurrence was defined as the lowest point of prolapse exceeding the level of the hymen.

### Statistical analysis

Normally distributed continuous variables are presented as the mean ± SD (standard deviation) and were analyzed using paired Student’s t test in the preoperative and follow-up period comparisons. Nonnormally distributed variables are described as the median (range), and the Wilcoxon rank-sum test was used to compare paired samples and the Mann‒Whitney U test for independent samples. Categorical variables are shown as numbers (percentages) and were analyzed using the chi-square test. A two-sided *P* < 0.05 was considered statistically significant. All statistical analyses were performed using STATA 13.0 (StataCorp, College Station, Texas, USA) and Prism GraphPad 7.0 (GraphPad Software, California, USA) software.

## Results

A total of 157 patients with stage III–IV POP received VALS in our hospital from January 2010 to December 2018. Among them, 22 patients were out of contact, and 29 patients refused to return. Finally, 106 patients completed the follow-up and were included in our analyses. All the following results were reported based on the last follow-up period point of each patient.

Table 1 shows the baseline demographic data of eligible patients. The mean age was 55.86 ± 8.48 years, body mass index (BMI) was 24.10 ± 2.89 kg/m², median parity was 2 (ranging from 1 to 6), and median follow-up period was 35.40 months (ranging from 12.13 to 109.97 months). Twenty-eight patients (26.42%) suffered from concomitant urinary incontinence, and 4 patients (3.77%) had a history of previous POP surgery. Fifty-nine patients (55.66%) were diagnosed with stage III POP, while the other 47 (44.34%) were diagnosed with stage IV. The detailed distribution of prolapse compartments for all patients is listed in Table 2.

**Table 1:** Baseline characteristics of included patients undergoing VALS

Variables	POP patients (n = 106)
Age, mean ± SD, years	55.86 ± 8.48
BMI, mean ± SD, kg/m²	24.10 ± 2.89
Gravidity, median (range)	3 (1–8)
Parity, median (range)	2 (1–6)
Menopause status, n (%)	76 (71.7)
*Comorbidity, n (%)*	
Hypertension	18 (16.98)
Diabetes	5 (4.72)
Coronary heart disease	3 (2.83)
Hyperthyroidism	2 (1.89)
Hypothyroidism	2 (1.89)
Hysteromyoma	26 (24.53)
Stress urinary incontinence	23 (21.70)
Urgent urinary incontinence	3 (2.83)
Mixed urinary incontinence	2 (1.89)
Depression	2 (1.89)
*Previous surgery history, n (%)*	
Tubal ligation	18 (16.98)
Cesarean delivery	4 (3.77)
Hysteromyomectomy	2 (1.89)
Pelvic reconstruction surgery	4 (3.77)
Vaginal repair	3 (2.83)
*POP-Q stage, n (%)*	
III	59 (55.66)
IV	47 (44.34)
Follow-up period, median (range), months	35.40 (12.13–109.97)

*VALS* Vaginally assisted laparoscopic sacrocolpopexy; *POP* Pelvic organ prolapse; *BMI* Body mass index; *SD* Standard deviation

**Table 2:** Prolapse compartment distribution of included patients

POP-Q stage	Anterior vaginal prolaose		Uterine prolapse		Posterior vaginal prolaose	
	n	Percent (%)	n	Percent (%)	n	Percent (%)
No prolapse	1	0.94	2	1.89	4	3.77
I	2	1.89	9	8.49	17	16.04
II	12	11.32	9	8.49	32	30.19
III	48	45.28	61	57.55	41	38.68
IV	43	40.57	25	23.58	12	11.32

*POP-Q* Pelvic organ prolapse quantification

In 106 patients receiving VALS, intra- and postoperative information is shown in Table 3. Laparoscopic hysterectomy was performed in 93 patients (87.74%), and the remaining 13 (12.26%) received vaginal hysterectomy. Concomitant operations included tension-free vaginal tape abbrevo (TVT-A) in 9 cases, tension-free vaginal tape obturator (TVT-O) in 5 cases and tension-free vaginal tape exact (TVT-E) in 6 cases. The mean operative time of hysterectomy plus sacrocolpopexy was 100.12 ± 20.05 min. The median estimated blood loss during surgery was 50 mL (ranging from 20 to 250 mL). No intraoperative complications occurred. Three patients had postoperative fever, and 1 patient suffered wound infection during hospitalization. The median hospitalization time after surgery was 5 days (ranging from 3 to 9 days), and urethral catheters were usually removed 2 days after the operation.

**Table 3:** Intra- and postoperative information of included patients

Variables	POP patients (n = 106)
*Concomitant surgery, n (%)*	
TVT-A	9 (8.49)
TVT-O	5 (4.72)
TVT-E	6 (5.66)
Perineal reconstruction	53 (50.00)
Vaginal wall repair	5 (4.72)
*Hysterectomy during surgery, n (%)*	
Laparoscopy	93 (87.74)
Vaginal	13 (12.26)
Hysterectomy + sacrocolpopexy time, mean ± SD, min	100.12 ± 20.05
Estimated blood loss, median (range), mL	50 (20–250)
Catheterization time after surgery, median (range), days	2 (1–7)
Hospitalization time after surgery, median (range), days	5 (3–9)

*POP* Pelvic organ prolapse; *TVT-A* Tension-free vaginal tape abbrevo; *TVT-O* Tension-free vaginal tape obturator; *TVT-E* Tension-free vaginal tape exact

**Fig. 2:**
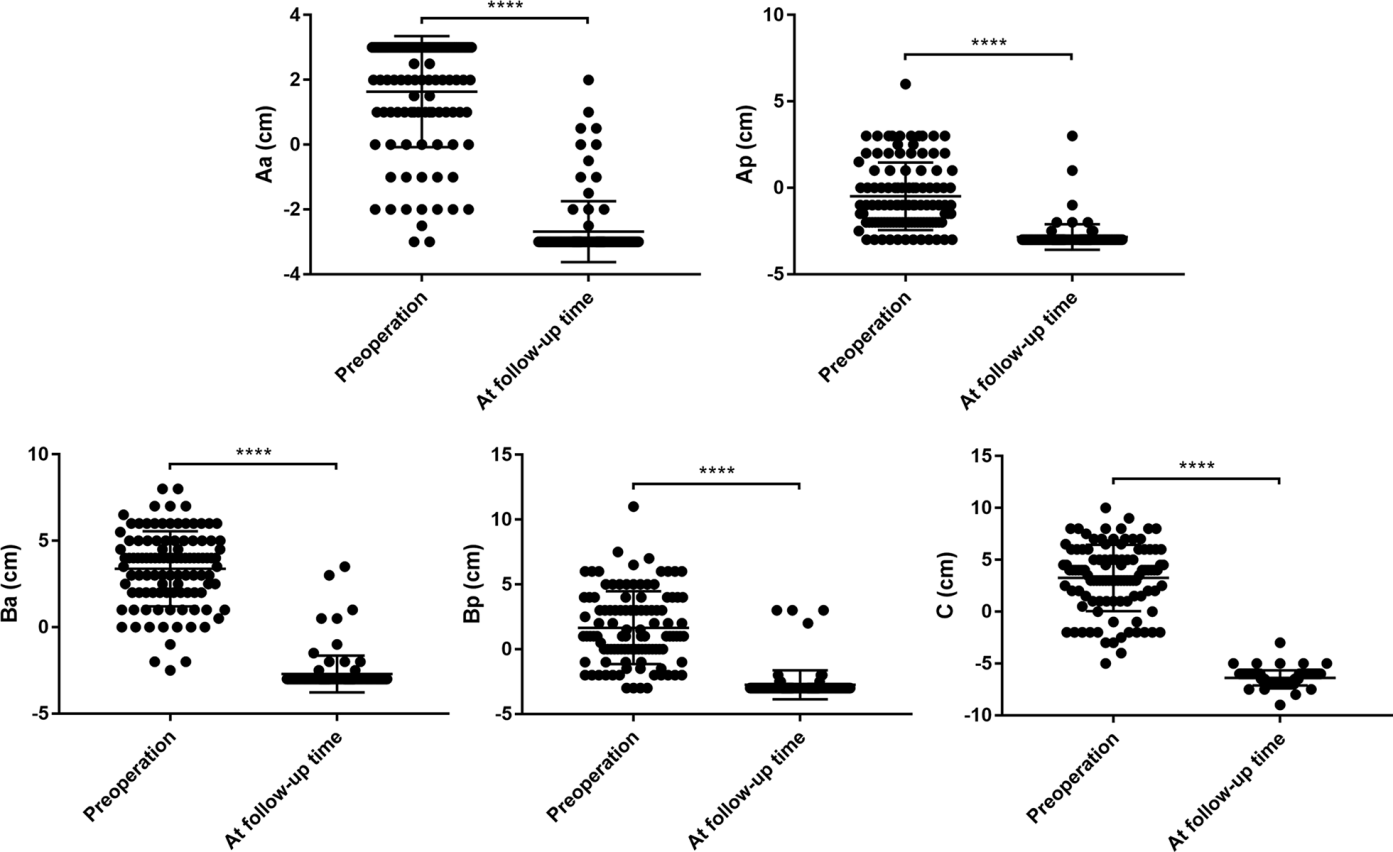
Comparisons of POP-Q points between preoperation and at follow-up (One dot represents one patient; ****: *P* < 0.0001)

All POP-Q points except pb at follow-up period significantly improved (*P* < 0.001) compared with preoperative values (Fig. 2; Table 4). During the follow-up period, 8 patients had recurrent prolapse, and the objective cure ratio reached 92.45% (98/106).

**Table 4:** Comparisons of POP-Q points between preoperation and at follow-up

POP-Q point	Preoperation, median (range), cm	At follow-up, median (range), cm	MD (95% CI)	*P* value
Aa	2.0 (− 3.0 to 3.0)	− 3.0 (− 3.0 to 2)	− 4.32 (− 4.66 to − 3.98)	< 0.001
Ba	4.0 (− 2.5 to 8.0)	− 3.0 (− 3.0 to 3.5)	− 6.09 (− 6.50 to − 5.68)	< 0.001
C	3.75 (− 5.0 to 10.0)	− 6.0 (− 9.0 to − 3.0)	− 9.63 (− 10.26 to − 9.00)	< 0.001
TVL	8.0 (6.0 to 10.0)	7.0 (3.5 to 9.0)	− 0.77 (− 0.98 to − 0.56)	< 0.001
Ap	− 1.0 (− 3.0 to 6.0)	− 3.0 (− 3.0 to 3.0)	− 2.36 (− 2.72 to − 2.00)	< 0.001
Bp	1.0 (− 3.0 to 11.0)	− 3.0 (− 3.0 to 3.0)	− 4.41 (− 4.98 to − 3.84)	< 0.001
gh	6.0 (2.0 to 10.0)	3.0 (2.5 to 7.0)	− 2.43 (− 2.70 to − 2.16)	< 0.001
pb	2.5 (1.0 to 6.5)	3.0 (2.0 to 4.0)	0.11 (− 0.06 to 0.27)	0.194

*POP-Q* Pelvic organ prolapse quantification; *MD* Mean difference; *CI* Confidence interval

**Fig. 3:**
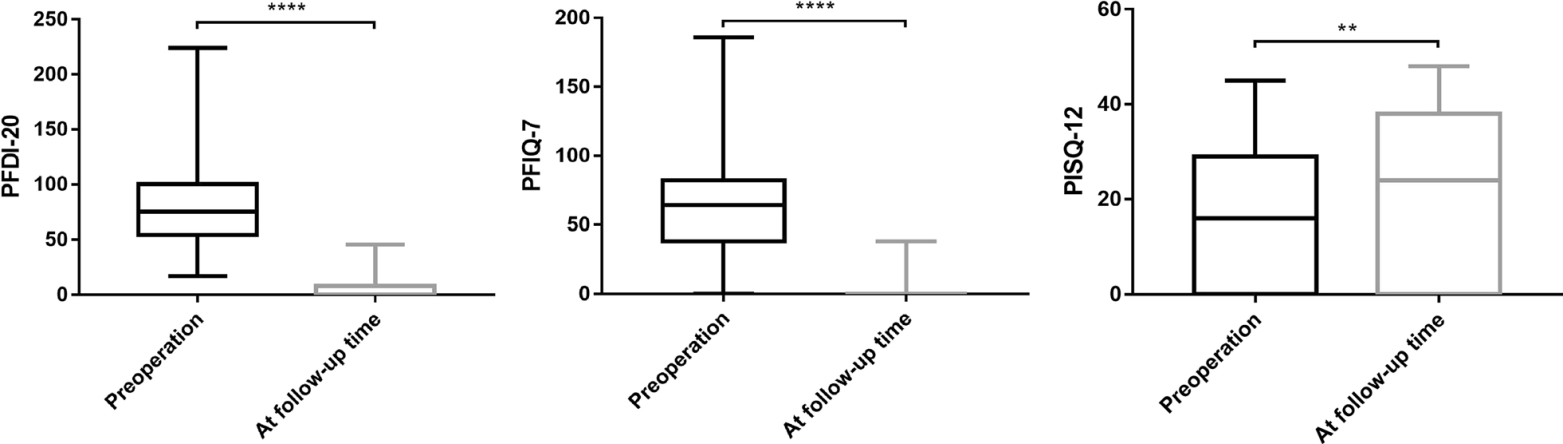
Comparisons of PFDI-20, PFIQ-7 and PISQ-12 scores preoperatively and at follow-up (**: *P* < 0.01, ****: *P* < 0.0001)

A total of 106 patients completed the PFDI-20, PFIQ-7 and PISQ-12 questionnaires before surgery and at follow-up. Patients’ subjective symptoms significantly improved [PFDI-20: mean of difference (MD) = − 72.76, 95% confidence interval (CI) − 78.97 to − 66.55, *P* < 0.001; PFIQ-7: MD = − 63.12, 95% CI − 69.13 to − 57.11, *P* < 0.001; PISQ-12: MD = 4.80, 95% CI 2.01–7.58, *P* = 0.0013] (Fig. 3; Table 5). For the PGI-I score, 99 (93.40%) patients’ responses were “very much improved”, 6 (5.66%) patients’ responses were “much improved”, and only 1 (0.94%) patient was “minimally improved”. Consequently, the subjective success rate was 99.06% (105/106).

**Table 5:** Subjective evaluation of included patients at follow-up period

Scales	Preoperation	At follow-up	MD (95% CI)	*P* value
PFDI-20, mean ± SD	78.72 ± 32.54	5.97 ± 11.02	− 72.76 (− 78.97 to − 66.55)	< 0.001
PFIQ-7, mean ± SD	63.88 ± 31.03	0.76 ± 4.63	− 63.12 (− 69.13 to − 57.11)	< 0.001
PISQ-12, mean ± SD	13.82 ± 14.73	19.59 ± 19.53	4.80 (2.01 to 7.58)	0.0013
PGI-C, n (%)				
Very much improved	–	99 (93.40)		–
Much improved		6 (5.66)		
Minimally improved		1 (0.94)		

*PFDI-20* Pelvic floor distress inventory-short form 20; *PFIQ-7* Pelvic floor impact questionnaire short form 7; *PISQ-12* Pelvic organ prolapse/urinary incontinence sexual questionnaire-12; *PGI-C* Patient global impression of improvement; *MD* Mean difference; *CI* Confidence interval

Detailed information on the 8 patients suffering anatomic prolapse recurrence is listed in Table 6. Six patients with relapsed anterior POP did not undergo surgery, while the other 2 patients with posterior POP underwent posterior pelvic reconstruction using Gynecare Prolift mesh. Thus, the reoperation rate of VALS was 1.89% (2/106).

**Table 6:** Details of patients suffering from POP recurrence

No.	Gravidity	Parity	BMI (kg/m²)	Follow-up period (months)	Preoperation POP-Q stage			Recurrence compartment	Recurrence POP-Q stage	Management for recurrence
					Anterior vaginal wall	Uterine	Posterior vaginal wall			
1	4	4	22.64	12.9	IV	IV	IV	Anterior vaginal wall	III	Conservative treatment
2	2	2	22.72	19.3	IV	IV	IV	Anterior vaginal wall	II	Conservative treatment
3	8	6	24.34	22.17	II	I	III	Posterior vaginal wall	III	Prolift posterior pelvic floor repair
4	5	5	26.78	25.43	IV	IV	IV	Posterior vaginal wall	III	Prolift posterior pelvic floor repair
5	2	2	27.94	25.6	III	III	II	Anterior vaginal wall	II	Conservative treatment
6	4	2	25.15	41.57	IV	I	I	Anterior vaginal wall	II	Conservative treatment
7	8	3	31.22	49.37	IV	IV	III	Anterior vaginal wall	II	Conservative treatment
8	3	2	22.49	61.73	IV	IV	IV	Anterior vaginal wall	II	Conservative treatment

*POP-Q* Pelvic organ prolapse quantification; *BMI* Body mass index

Table 7 shows the VALS complications of the included patients. In 8 patients (7.55%) with late complications, there were 6 cases (5.66%) of mesh exposure on the vaginal wall that were treated via surgical incision of exposed meshes. De novo urinary incontinence occurred in 2 cases (1.89%), and anti-incontinence operations were subsequently performed. No patients complained of pain during intercourse.

**Table 7:** Complications of included patients after surgery

Variables	POP patients (n = 106)
*Early complications, n (%)*	
Fever	3 (2.83)
Wound infection	1 (0.94)
*Late complications, n (%)*	
Mesh exposure	6 (5.66)
De novo urinary incontinence	2 (1.89)

*POP* Pelvic organ prolapse

## Discussion

In this study, we analyzed the efficacy and safety of VALS for treating 106 patients with stage III–IV POP with a median follow-up period of 35.4 months. We found that the objective cure rate of VALS was 92.45%, and the subjective success rate reached 99.06%. No intraoperative complications occurred. The postoperative infection rate was 0.94%, and the mesh exposure rate was 5.66% during the follow-up period. We first reported the medium- to long-term efficacy and safety of VALS for treating patients with stage III–IV POP, and our results indicated that VALS was effective and safe.

In our study, we excluded posthysterectomy patients. Dissection of the anterior vaginal wall is performed through vaginal incision left by hysterectomy but not through a longitudinal incision on the vaginal wall. The vaginal residual already healed in patients who received hysterectomy before, so a transvaginal mesh requires an extra incision on the vaginal wall, which will undoubtedly cause higher infection and mesh exposure rates. For these patients, we chose a single laparoscopic operation instead of a transvaginal operation. In Tapisiz et al.’s study, a case who received VALS with retroperitoneal tunneling was introduced [18]. They summarized this operation in their review and concluded that patients who desired uterine preservation preferred this method to achieve better efficacy and shorter operative time [19].

For III–IV POP females, multicompartment at different levels is often observed, which is appropriate to be corrected in one LSC [20]. However, conducting operations in the deep pelvis takes great difficulties, including tissue dissection, suturing and mesh placement [14]. Proficient manipulations and rich experiences are undoubtedly necessary for successful LSC, while LSC also requires a longer operative time with more blood loss. Therefore, VALS occurred to cover the shortage of LSC by directly reaching the deep operative target via the vagina. In our opinion, VALS has advantages of easier tissue dissection and shorter operative time than LSC, which is in line with the study by Aydin et al. [8]. Thus, VALS may be more suitable for patients who are not tolerant of long-term operation. Although TVM also requires a shorter operative time, it should be very carefully applied due to the vaginal synthetic mesh warnings raised by the FDA [4].

Although seldom reported in published literature, VALS demonstrated promising efficacy in short-term postoperative follow-up. Athanasiou et al. conducted a prospective pilot study on VALS for severe POP in 2012. In a 12 month observation of 27 women, a 100% success rate was achieved, and subjective outcomes proved to be satisfactory [14]. Aydin and colleagues recently compared VALS with traditional abdominal sacrocolpopexy. During a mean follow-up period of 20 months, the objective failure rate, subjective failure rate and recurrence rate remained almost similar in both groups [8]. We previously investigated a cohort of 65 severe POP patients receiving VALS and followed them up to approximately 24 months, concluding that all POP-Q scores improved except pb. In addition, postoperative PFDI-20, PFIQ-7, and PISQ-12 scores all turned out to be significantly different compared with preoperative values [21]. In our study, we enlarged the cohort to 106 cases, including III and IV POP, and extended the follow-up period to an average of 35.4 months. Promising efficacy was finally observed, with a 92.45% objective cure rate and a 99.06% subjective cure rate. Eight patients suffered from recurrence. Six of them (75%) had anterior prolapse. This recurrence rate was obviously lower than that in the Miedel et al. [22] and Liu et al.’s [23] studies, in which LSC was applied. Anterior prolapse is the most common type of postoperative POP recurrence. Theoretically, mesh can be placed in a more accurate lower position in the deep pelvis by VALS, so we gained a relatively low recurrence rate of anterior prolapse. Larger sample sizes in planned comparative studies are expected to further confirm this result.

Complications of vaginal procedures always attract many concerns. A previous publication mentioned a higher wound infection rate when surgical procedures were performed vaginally [24]. In our study, only 1 infection case (0.94%) was found in the early postoperative follow-up period. We carried out a series of standard preoperative precautions to prevent the infection, including the use of a pessary, topical estrogen ointment and potassium permanganate sitz bath. The above strategies may decrease the infection risk brought about by transvaginal wounds.

Mesh erosion requires attention in late complications, which causes great pain and decreases patients’ quality of life. An increased mesh erosion rate was considered a weakness of VALS compared to LSC because of the transvaginal placement of the mesh [9]. However, the mesh erosion rate was acceptable (5.66%) in our study compared with that reported for abdominal SC [25]. A series of measures were taken by our team to avoid erosion to the greatest extent. Perioperative vaginal use of estrogen (1 week before operation and 3 months after operation) benefited vaginal wound healing because most patients receiving our VALS were in perimenopause [26]. Lack of experience was a significant risk factor for mesh erosion [27, 28]. All our included patients underwent VALS by senior skilled surgeons, and our team has published several successful cohorts, accumulating adequate experience with VALS.

According to the literature, VALS has a shorter operation and hospitalization time. It is easier for junior surgeons to handle vaginal manipulations instead of vesicovaginal and rectovaginal space dissection through laparoscopy [8, 29]. Furthermore, compared with robot-assisted operations, VALS is economical and still holds the position in POP surgical treatments [30]. More evidence needs to be raised by comparative studies and systematic reviews.

Although our study was carried out as a case series, it had some strengths. First, all previous studies reporting VALS had a very limited number of patients, while our sample size was relatively large. Second, except for objective outcomes, we also evaluated the subjective outcomes of patients using several questionnaires, which have rarely been reported by other studies on VALS. Third, the follow-up period of our study was longer than that of previous studies. All the strengths above may be helpful to benefit the current understanding of VALS.

Our study also had several limitations. First, the percentage of patients who completed follow-up was relatively low (67.52%, 106/157); thus, the sample size still needs to be enlarged, and a prospective study is necessary. Second, the heterogeneity of surgical procedures existed in our case series. Although all patients received hysterectomy and VALS, some patients also received different concomitant operations, such as TVT. We conducted TVT on patients with severe urinary incontinence because mild to moderate urinary incontinence could be relieved after POP was corrected by VALS. Therefore, selection bias was unavoidable owing to the choice of VALS itself and different detailed surgical manipulations. Third, the POP-Q test was performed by the surgeon, which may also lead to bias due to unblinded assessment. Finally, all surgeries were performed by senior skilled surgeons in our study, which limited its reference value for junior surgeons.

## Conclusion

VALS is an effective and safe procedure for treating severe POP. Therefore, VALS operations should be considered for treating severe POP due to its favorable subjective and objective outcomes, low rate of infection and acceptable rate of mesh exposure.

## Data Availability

All relevant data and materials of this study were included within this published article.

## Abbreviations

BMI: Body mass index
FDA: Food and Drug Administration
LSC: Laparoscopic sacral colpopexy
MD: Mean of difference
PFDI-20: Pelvic floor distress inventory-short form 20
PFIQ-7: Pelvic floor impact questionnaire short form 7
PGI-I: Patient global impression of improvement
PISQ-12: Pelvic organ prolapse/urinary incontinence sexual questionnaire-12
POP: Pelvic organ prolapse
POP-Q: Pelvic organ prolapse quantification
SD: Standard deviation
TVM: Transvaginal mesh
TVT-A: Tension-free vaginal tape abbrevo
TVT-E: Tension-free vaginal tape exact
TVT-O: Tension-free vaginal tape obturator
VALS: Vaginally assisted laparoscopic sacrocolpopexy
